# Mobile vaccination units to increase COVID-19 vaccination uptake in areas with lower coverage: a within-neighbourhood analysis using national registration data, the Netherlands, September–December 2021

**DOI:** 10.2807/1560-7917.ES.2024.29.34.2300503

**Published:** 2024-08-22

**Authors:** Mattijs S Lambooij, Joyce Pijpers, Jan van de Kassteele, Mirjam P Fransen, Susan JM Hahné, Niek Hof, Floor M Kroese, Hester de Melker, Mart van Dijk, Ellen Uiters, Marijn de Bruin

**Affiliations:** 1 Centre for Prevention, Lifestyle and Health, Department Behaviour & Health, National Institute for Public Health and the Environment (RIVM), Bilthoven, the Netherlands; 2 Erasmus University, Erasmus School of Health Policy and Management, Rotterdam, the Netherlands; 3 Centre for Epidemiology and Surveillance of Infectious Diseases, National Institute for Public Health and the Environment (RIVM), Bilthoven, the Netherlands; 4 Department of Statistics, Data Science and Mathematical Modelling, National Institute for Public Health and the Environment (RIVM), Bilthoven, the Netherlands; 5 Amsterdam UMC, University of Amsterdam, Department of Public and Occupational Health, Amsterdam Public Health Research Institute, Amsterdam, the Netherlands; 6 Dutch National Coordination for COVID-19 Control, The Hague, the Netherlands; 7 Utrecht University, Department of Social, Health and Organizational Psychology, Utrecht, the Netherlands; 8 Centre for Food, Prevention and Health Care, National Institute for Public Health and the Environment (RIVM), Bilthoven, the Netherlands; 9 Radboud University Medical Centre, Institute of Health Sciences, IQ Health, Nijmegen, the Netherlands

**Keywords:** Public health, vaccination, inequality, intervention, effect evaluation, mobile vaccination units

## Abstract

**Background:**

Vaccine uptake differs between social groups. Mobile vaccination units (MV-units) were deployed in the Netherlands by municipal health services in neighbourhoods with low uptake of COVID-19 vaccines.

**Aim:**

We aimed to evaluate the impact of MV-units on vaccine uptake in neighbourhoods with low vaccine uptake.

**Methods:**

We used the Dutch national-level registry of COVID-19 vaccinations (CIMS) and MV-unit deployment registrations containing observations in 253 neighbourhoods where MV-units were deployed and 890 contiguous neighbourhoods (total observations: 88,543 neighbourhood-days). A negative binomial regression with neighbourhood-specific temporal effects using splines was used to study the effect.

**Results:**

During deployment, the increase in daily vaccination rate in targeted neighbourhoods ranged from a factor 2.0 (95% confidence interval (CI): 1.8–2.2) in urbanised neighbourhoods to 14.5 (95% CI: 11.6–18.0) in rural neighbourhoods. The effects were larger in neighbourhoods with more voters for the Dutch conservative Reformed Christian party but smaller in neighbourhoods with a higher proportion of people with non-western migration backgrounds. The absolute increase in uptake over the complete intervention period ranged from 0.22 percentage points (95% CI: 0.18–0.26) in the most urbanised neighbourhoods to 0.33 percentage point (95% CI: 0.28–0.37) in rural neighbourhoods.

**Conclusion:**

Deployment of MV-units increased daily vaccination rate, particularly in rural neighbourhoods, with longer travel distance to permanent vaccination locations. This public health intervention shows promise to reduce geographic and social health inequalities, but more proactive and long-term deployment is required to identify its potential to substantially contribute to overall vaccination rates at country level.

**Key public health message**

**What did you want to address in this study and why?**

During the COVID-19 pandemic, the local health services in the Netherlands deployed mobile vaccination units in neighbourhoods where not many people were getting vaccinated. We aimed to study whether these mobile units helped increase vaccination rates in those areas and specify areas where deployment was most beneficial.

**What have we learnt from this study?**

In the most urban areas, twice as many got vaccinated when mobile vaccination units were placed in their neighbourhood. In rural areas, the increase was a factor 14.5. The increase was more noticeable in neighbourhoods with > 5% voting for the Dutch conservative Reformed Christian party, and less in neighbourhoods with a higher percentage of people with a non-western migration background.

**What are the implications of your findings for public health?**

We recommended to deploy mobile vaccination units in neighbourhoods where vaccine uptake is lagging, especially in rural areas. Hereby, it is important to ensure that a healthcare worker who can answer questions and provide additional information is present.

## Introduction

Willingness to be vaccinated is affected by individual, social, organisational and vaccine-specific factors [1]. Both willingness to be vaccinated and actual vaccine uptake vary significantly across different social groups. Even in countries with relatively low inequality, such as Finland, socioeconomic aspects affect vaccine uptake [2]. Reducing inequality in vaccine uptake may be considered an effective means to reduce health inequalities between social groups. Vaccination against severe acute respiratory syndrome coronavirus 2 (SARS-CoV-2) (hereinafter called COVID-19 vaccine) has proven highly effective in mitigating severe illness and reducing mortality caused by the virus [3]. Demographic factors such as age, education level, ethnicity and geographical factors (e.g. degree of urbanisation) are associated with differences in vaccine uptake [1,4,5]. As low vaccine uptake tends to cluster socioeconomically or spatially [6,7], effective interventions to reduce inequality may teach valuable lessons to reduce overall inequity in health. During the COVID-19 pandemic, mobile vaccination units (MV-units) were deployed in the Netherlands, like in other countries [6,7], to specifically target neighbourhoods with lower vaccine uptake.

In the Netherlands, the primary COVID-19 vaccination campaign started in January 2021. The vaccines were rolled out in a staggered fashion, first the oldest people and people with a high risk due to chronic conditions were offered vaccination, followed by subsequent younger age groups. Within the oldest age group (> 90 years), 89% had received at least one COVID-19 vaccine and 87% had received the complete primary vaccination set of two doses on 1 September 2021 [8]. Vaccine uptake was lower among the youngest age group (18–25 years): 67% had received at least one COVID-19 vaccine and 57% the complete set of two doses [8]. From September to October 2021, the increase in vaccine uptake had levelled off and, only a very limited number of people came to vaccination locations to receive their first dose. Besides differences in age, the uptake differed between neighbourhoods. Recognising these disparities, the Dutch municipal health services strategically deployed mobile vaccination units (MV-units) in neighbourhoods with lower vaccine uptakes guided by regional vaccination registration and knowledge of hard-to-reach population segments. Typically, these targeted neighbourhoods exhibited a higher proportion of individuals with (i) lower educational attainment, (ii) a non-western migration background and (iii) > 5 % of voters for the Reformed Political Party (RPP, Dutch: Staatkundig Gereformeerde Partij, a conservative Reformed Christian political party) [8-10]. In addition, lower vaccine uptakes were seen in the most urbanised areas of the Netherlands [8-10].

The purpose of the MV-units (either as buses or temporal pop-up locations) was to facilitate vaccination by improving the physical accessibility of vaccination services and easy accessibility to healthcare professionals to answer vaccine-related questions. The MV-units were equipped with enough vaccines, registration facilities and a waiting room. This was supported by communication and information provision. Poster, flyers, flags and social media were used to announce the presence and oftentimes healthcare professionals (doctors, nurses) were on site to answer questions. The 25 Dutch municipal health service regions and each location were autonomous in the forms of support, resulting in different strategies per region. In practice, this meant that in neighbourhoods with a higher proportion of people with lower educational levels, with more people with non-western migration backgrounds or higher percentage of voters for the RPP, the municipal health services cooperated with local key figures, other than healthcare professionals. These key figures differed per region and could be religious leaders but also social workers or volunteers.

To date, no studies have been conducted investigating the effect of the deployment of MV-units in the Netherlands. In the United Kingdom (UK), the vaccine uptake in nine regions increased by 25% when deploying MV-units [11], and in the United States (US), MV-units increased vaccination rates among groups with lower uptake than average [12]. Therefore, the aim of this study was to investigate whether and, if so, to which extent, the deployment of MV-units was effective in increasing uptake of COVID-19 vaccine in neighbourhoods.

## Methods

### Study design

We used a natural-experimental design to assess the effect of the deployment of MV-units on daily vaccination rate and vaccine uptake. Unlike a randomised controlled trial, where participants are randomly assigned to intervention or control groups, the deployment of the MV-units was determined by regional health services based on low vaccination coverage compared with other neighbourhoods in their region. As a result, the deployment of the MV-units varied over time and over neighbourhood. An overview of the deployment of the MV-units is shown in Supplementary Figure S3. We used this variation to study whether and to which extent the number of daily administered vaccines significantly differed from periods when MV-units were not deployed.

### Study setting and population

The study population comprised neighbourhoods within 16 of 25 municipal health regions in the Netherlands. The 16 municipal health regions comprised a total of 1,183 neighbourhoods. For administrative use by municipalities and data collection by the Statistics Netherlands (CBS), all municipalities are subdivided into districts, which in turn are subdivided into neighbourhoods. Districts and neighbourhoods have no formal status. Districts and neighbourhoods are coherent areas that are based on several characteristics like age, geographical barriers such as busy roads, having similar urban and/or architectural features or having similar functional, social or political characteristics. The MV-units were present in 256 neighbourhoods for at least 1 day. Three units were placed at one location for a longer period: 38, 57 and 90 consecutive days. We considered these to be non-incidental vaccination units and these neighbourhoods were therefore excluded for this analysis. In the remaining 253 neighbourhoods, most units were present for only 1 day and for no more than six consecutive days in all neighbourhoods.

#### Contiguous neighbourhoods

The MV-units served all people who wished to receive a COVID-19 vaccine, regardless of which neighbourhood people lived in. This means that inhabitants of contiguous neighbourhoods could also be reached across the borders of the neighbourhood where the unit was stationed. This is called a spill-over effect [13]. We defined a contiguous or spill-over neighbourhood as a neighbourhood that shares a border with a neighbourhood where a MV-unit was present, here called targeted neighbourhood. We included 253 targeted neighbourhoods and 890 contiguous neighbourhoods. Because of overlapping targeted and non-targeted neighbourhoods, the total number of neighbourhoods included for analyses was 973. Table 1 presents information on relevant characteristics of included and excluded neighbourhoods.

**Table 1:** Descriptive statistics of neighbourhoods included and excluded in a study on the effect of mobile vaccination units on COVID-19 vaccine uptake, the Netherlands, September–December 2021

Characteristics	Population size	Vaccine uptake (%)	People with migration background (%)	Voters for the RPP (%)
Mobile vaccination unit deployed				
Not urbanised	135,589	61.0	3.62	10.9
Hardly urbanised	446,598	64.1	5.28	6.40
Moderately urbanised	452,112	64.5	9.56	3.72
Strongly urbanised	1,246,298	60.9	19.5	1.55
Extremely urbanised	778,406	51.9	40.6	0.61
Mobile vaccination unit not deployed				
Not urbanised	1,174,951	67.9	3.02	3.88
Hardly urbanised	1,255,181	68.3	5.01	2.80
Moderately urbanised	1,453,003	65.0	10.2	2.73
Strongly urbanised	2,448,989	63.3	15.0	1.71
Extremely urbanised	2,528,944	58.5	26.8	0.65

RPP: Reformed Political Party (in Dutch: Staatkundig Gereformeerde Partij, a conservative Reformed Christian political party).

Not urbanised (< 500 addresses/km^2^); hardly urbanised (500–999 addresses/km^2^); moderately urbanised (1,000–1,499 addresses/km^2^); strongly urbanised (1,500–2,499 addresses/km^2^); extremely urbanised (> 2,5000 addresses/km^2^).

### Outcome measures

In this study, two outcome measures were used. Firstly, the vaccination rate, defined as the daily number of individuals that received a vaccination against COVID-19 divided by the number of eligible people in that neighbourhood. Secondly, the vaccine uptake, defined as the proportion of individuals in a neighbourhood who received at least one dose of COVID-19 vaccination. Vaccination data were retrieved from the COVID-19 Vaccination Information and Monitoring System (CIMS). The CIMS database includes data of people who gave informed consent to register their vaccination data (93%).

### Covariates

The level of urbanisation, socioeconomic status (SES), the presence of people with a non-western migration background and voters for the RPP have been associated with vaccine uptake [9]. Therefore, data on the level of urbanisation, socioeconomic status score (based on income, assets, education and recent labour activity [14], percentage of people with a non-western migration background and percentage of voters for the RPP [15,16] in the neighbourhoods were included in the analysis. Historically, lower vaccination coverage regarding vaccines included in the national immunisation programme (NIP) have been seen among people practising a Reformed Protestant faith in the Netherlands [17]. As we did not have data on church membership or other individual-level data on religion, we used the percentage of votes for the RPP as a proxy variable for people practising the Reformed Protestant faith, as has been done in previous epidemiological studies of the Dutch context [18,19]. Areas with a high percentage of people practising the Reformed Protestant faith, also referred to as the Dutch Bible Belt, are defined by areas with > 5% of the votes for the RPP. Results of these studies indicate strong associations between the proxy variable and vaccination outcomes [18,19]. The level of urbanisation was divided into five categories: not urbanised (< 500 addresses/km^2^); hardly urbanised (500–999 addresses/km^2^), moderately urbanised (1,000–1,499 addresses/km^2^), strongly urbanised (1,500–2,499 addresses/km^2^) and extremely urbanised (≥ 2,500 addresses/km^2^).

### Descriptive analysis

We created a geographical map to present vaccine uptake per neighbourhood on 1 September 2021 (S0). Furthermore, we created another map showing the placement of MV-units in the 973 neighbourhoods included in the study between 1 September and 1 December 2021. The map is shown in Supplementary Figure S2. The total number observations was 88,543 neighbourhood-days.

### Statistical analysis

Because of the quasi-experimental design, we formulated expectations about the causal relationships between the presence of MV-units and the daily vaccine uptake, before designing the regression model. Date, weekday, vaccine uptake and the neighbourhood itself were identified as confounding variables. The graph of the expected causal pathways is shown in Supplementary Figure S1, presenting the minimal sufficient adjustment set for estimating the total effect of the presence of the MV-units on the vaccination rate including the confounding variables [20]. The main advantage of this model is that neighbourhoods serve as controls of themselves. Consequently, all factors related to neighbourhoods are incorporated in the analyses.

#### Regression analysis

##### Effect of mobile vaccination units on daily vaccination rate

We formulated a negative binomial generalised linear mixed model to describe the daily number of people that received a vaccination in a neighbourhood as a function of combinations of variables of the minimal adjustment set. We specified a neighbourhood specific smooth temporal effect using a spline construct and neighbourhood specific day-of-the-week effect to describe the daily baseline vaccine uptake in a neighbourhood. The effect of the presence of a MV-unit is modelled as a neighbourhood specific deviation from this baseline uptake. Similar terms were added for spill-over effects. In pseudo-equation form the model can be written as follows:^†^


$$V_{t,n}\;\sim \;NegBin\left(\mu_{t,n},\theta \right)$$

$$\log\left(\mu_{t,n}\right)=\log\left({pop}_{t,n}\right)+\log\left(\lambda_{t,n}\right)$$

$$log\left(\lambda_{t,n}\right)=constant$$

$$+\;s\left({date}_{t}\right)+re\left({neigh}_{n}\right)+s\left({day}_{t}\right)$$

$$+\;s\left({date}_{t},{neigh}_{n}\right)+re\left({day}_{t},{neigh}_{n}\right)$$

$$+\;{unitpresent}_{t,n}+re\left({unitpresent}_{t,n},{neigh}_{n}\right)$$

$${+\;unitspillover}_{t,n}+re({unitspillover}_{t,n},{neigh}_{n})$$

Here, *V_t,n_* represented the daily number of people that received a vaccination at date *t* in neighbourhood *n*, *µ_t_*_,_*_n_* the expected number of daily vaccinations and *θ* the overdispersion parameter of the negative binomial distribution. This parameter was treated as a nuisance parameter, log(*pop_t,n_*) was the offset term and represented the number of persons eligible for vaccination, *λ_t_*_,_*_n_* was the expected vaccination rate. Term s(*date_t_*) represented the overall smoothly varying time effect, modelled as a penalised spline (s), re(*neigh_n_*) represented a neighbourhood-specific random effect (re) and re(*day_t_*) a periodic day-of-the-week random effect. The time effect and day-of-the-week effect were allowed to vary between neighbourhoods by the s(*date_t_*, *neigh_n_*) and re(*day_t_*, *neigh_n_*) terms, respectively. The *unit-present_t_*_,_*_n_* term represented effect of the presence of a MV-unit in a hypothetical average neighbourhood. This so-called average neighbourhood embodied the average characteristics of the population studied. This effect was allowed to vary between neighbourhoods by the re*(unit-present_t,n_, neigh_n_*) random effect term, resulting in heterogeneous treatment effects. Two similar terms were included for possible spill-over effects to contiguous neighbourhoods. To reduce complexity of the model, the effect of MV-units was held constant over time.

The model appeared to be too complex to be fitted to all neighbourhoods together. Therefore, we stratified the analyses into quintiles of the level of urbanisation, because it could be expected that neighbourhoods in these strata had similar characteristics. We could have chosen any other variable to stratify on, since each neighbourhood serves as its own control.

The effects of the deployment of MV-unit in a neighbourhood on the daily vaccination rate are presented in the form of relative rates (RR), compared with the same neighbourhood with no MV-unit deployed (baseline).

##### Effect of mobile vaccination units on vaccine uptake

Assuming that people who chose to get vaccinated in a MV-unit would not have been vaccinated otherwise, the regression model can be used to estimate the increase in vaccine uptake caused by the deployment of MV-units.

The eventual vaccine uptakes in case MV-units had not been deployed, could be found by setting the effect sizes of the MV-units for each neighbourhood to zero for both the targeted and contiguous neighbourhoods. For each date and neighbourhood, expected vaccination rates *λ*^1^*_t_*_,_*_n_* and *λ*^0^*_t_*_,_*_n_* were calculated with deployment and without deployment of MV-units, respectively. These rates can be considered as hazard rates. There is a direct relation between the cumulative hazard rate Λ and the proportion of individuals that have encountered an event, here, in this context, getting vaccinated. The vaccine uptake *U_n_* in neighbourhood *n* at the end of the study period at *t* = 91 was calculated as follows: *U_n_* = 1 − exp(-*Λ_n_*), where *Λ_n_* = *Λ_t_* _= 0,_*_n_* + Σ*_t_* _= 1..91_ *λ_t_*_,_*_n_*. Here, *Λ_t_* _= 0,_*_n_* was the cumulative hazard rate at the beginning of the study period, calculated from the given vaccine uptake at *t* = 0. The effect size for each neighbourhood was calculated as the difference *U*^1^*_n_ – U*^0^*_n_*. Because the relation between the vaccine uptake and the vaccination rates was nonlinear, 95% confidence intervals (CI) were calculated by Monte Carlo simulation of the estimated regression model parameters.

The effects of the deployment of MV-unit in a neighbourhood on the eventual vaccine uptake are presented in the form of absolute risk differences (RD, expressed a percentages), compared with the same neighbourhood with no MV-unit deployed at all.

All analyses were carried out in R [21]. The regression model was fitted using the bam function from the mgcv package [21-23]. The pooled estimates are based on random-effect meta-analyses.

## Results

### Descriptive analysis

**Figure 1:**
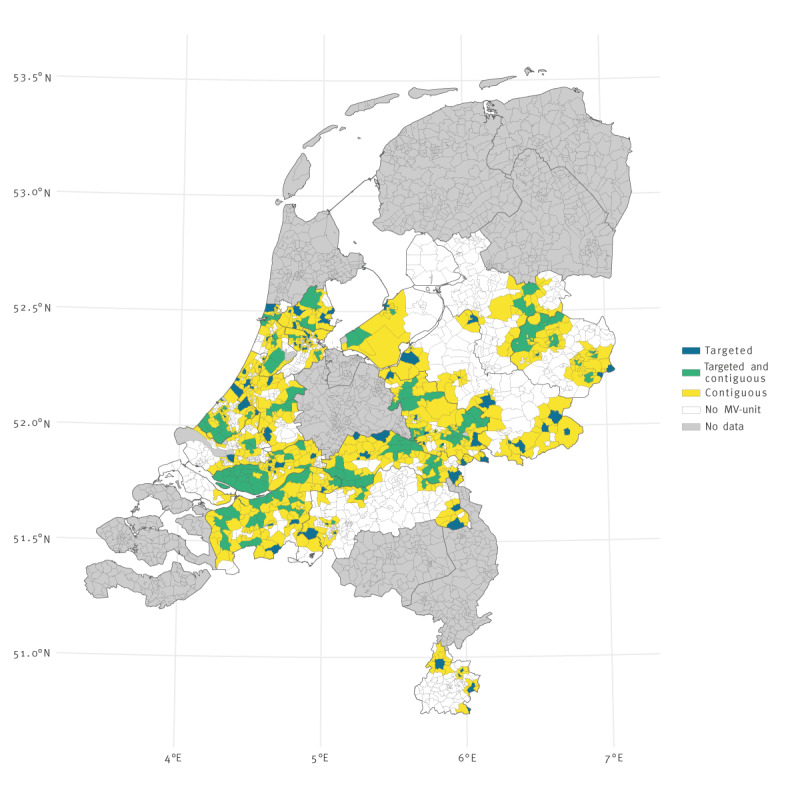
Map showing the placement of mobile vaccination units for COVID-19 vaccine in targeted neighbourhoods for at least 1 day, the Netherlands, September−December 2021 (n = 973)^a^

Figure 1 shows a map of the presence of MV-units for at least 1 day between 1 September and 1 December 2021, in 16 of the 25 municipal health regions. The duration of the presence of a MV-unit varied considerably. In most neighbourhoods, the MV-units were deployed on multiple occasions, but only for 1 day each time. More detailed information on the presence of MV-units by date and neighbourhood can be found in Supplementary Figure 3.

MV-unit: mobile vaccination unit.

^a^ 16 of the 25 municipal health regions were included.

The blue-coloured and green-coloured areas are neighbourhoods where a unit was present. The green-coloured regions were also indicated as contiguous neighbourhoods during this period. The yellow-coloured regions are contiguous neighbourhoods where no unit was present. The thick grey lines indicate the boundaries of the 25 municipal health regions in the Netherlands.

#### Regression analysis, impact of mobile vaccination units

In an average targeted neighbourhood where a MV-unit was deployed, during deployment, the daily vaccination rate in for example a moderately urbanised neighbourhood was a factor 5.7 (95% CI: 4.9–6.8) higher compared with the same neighbourhood when a unit was not deployed (Table 2). In an average neighbourhood, the spill-over effect in for example a moderately urbanised neighbourhood resulted in a factor 2.0 higher daily vaccination rate.

**Table 2:** Pooled relative rates of daily vaccination rates against COVID-19 on a day with and without a deployed mobile vaccination unit in a neighbourhood, stratified by level of urbanisation, the Netherlands, September–December 2021

Characteristics	Relative daily vaccination rate	95% CI
Targeted neighbourhoods^a^		
Not urbanised	14.5	11.6–18.0
Hardly urbanised	8.4	7.3–9.6
Moderately urbanised	5.7	4.9–6.8
Strongly urbanised	3.8	3.4–4.2
Extremely urbanised	2.0	1.8–2.2
Contiguous neighbourhoods^b^		
Not urbanised	2.7	2.6–2.8
Hardly urbanised	1.8	1.7–2.0
Moderately urbanised	2.0	1.9–2.2
Strongly urbanised	1.8	1.7–1.8
Extremely urbanised	1.4	1.3–1.5

CI: confidence interval.

^a^ Neighbourhood where a mobile vaccination unit was deployed.

^b^ Neighbourhood that shares a border with a targeted neighbourhood.

Not urbanised (< 500 addresses/km^2^); hardly urbanised (500–999 addresses/km^2^); moderately urbanised (1,000–1,499 addresses/km^2^); strongly urbanised (1,500–2,499 addresses/km^2^); extremely urbanised (≥ 2,500 addresses/km^2^).

**Figure 2:**
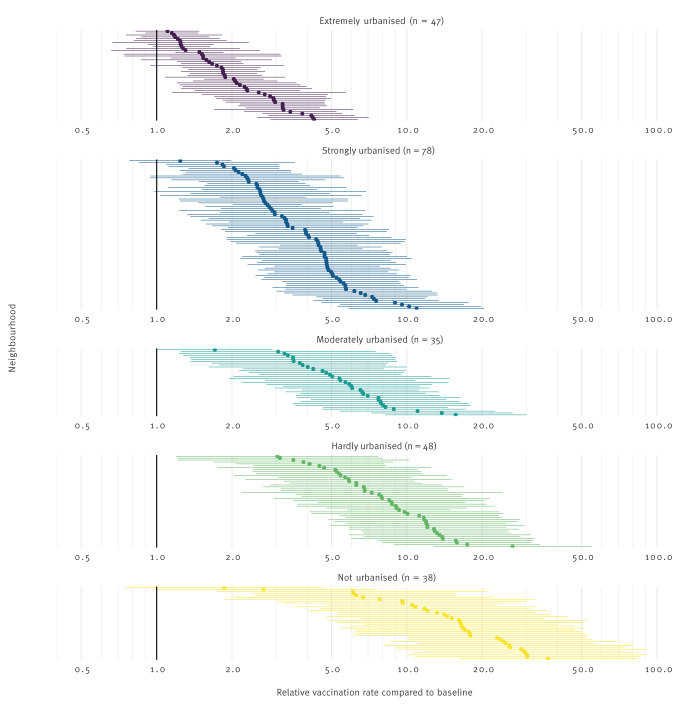
Relative rates of daily vaccination rates against COVID-19 on a day with and without a mobile vaccination unit in a neighbourhood, stratified by level of urbanisation, the Netherlands, September–December 2021

In the neighbourhoods where a unit was present, we found large differences in effect sizes between neighbourhoods (1.1–35.8) (Figure 2). In the contiguous neighbourhoods, the effect size varied between 0.8 and 6.8. Effect sizes decreased with an increasing level of urbanisation, as shown in Supplementary Figure S5.

The figure presents the relative rates and their 95% confidence intervals by neighbourhood.

Not urbanised (< 500 addresses/km^2^); hardly urbanised (500–999 addresses/km^2^); moderately urbanised (1,000–1,499 addresses/km^2^); strongly urbanised (1,500–2,499 addresses/km^2^); extremely urbanised (≥ 2,500 addresses/km^2^).

**Figure 3:**
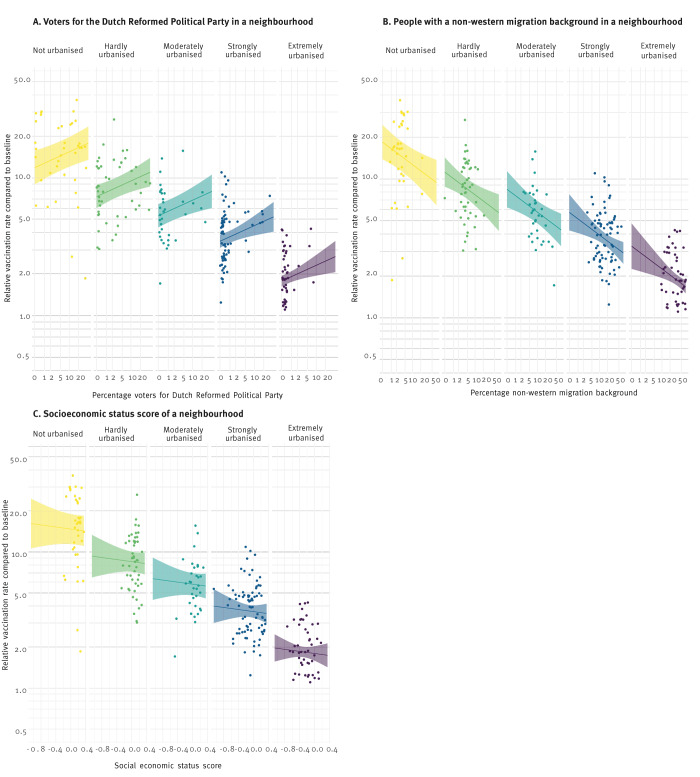
Effect of deployed mobile vaccination units on relative vaccination rates in neighbourhoods by percentage of voters for the Dutch Reformed Political Party (A), percentage of people with a non-western migration background (B) and socioeconomic status score (C), stratified by level of urbanisation, the Netherlands, September–December 2021 (n = 252 targeted neighbourhoods)

RPP: Reformed Political Party (in Dutch: Staatkundig Gereformeerde Partij, a conservative Reformed Christian political party); RR: relative rate.

Because the effects of the deployment of MV-units were very heterogeneous, we further investigated which neighbourhood characteristics were associated with these different effect sizes. Figure 3 shows the estimated effect sizes RR in relation to a selection of neighbourhood characteristics: percentage of votes for the RPP, SES and percentage of people with a non-western migration background. The association between SES and vaccine uptake was explained by the level of urbanisation in the neighbourhood (Figure 3C), in areas where more people voted for the RPP, the effect of the MV-unit was stronger, and in areas with more people with a non-western migration background, the effect was smaller (Figures 3A and B).

The panels show the estimated effect sizes RR (dots) in relation to a selection of corresponding neighbourhood characteristics. The lines represent the estimated regression lines. Note: some variables have been transformed.

Not urbanised (< 500 addresses/km^2^); hardly urbanised (500–999 addresses/km^2^); moderately urbanised (1,000–1,499 addresses/km^2^); strongly urbanised (1,500–2,499 addresses/km^2^); extremely urbanised (≥ 2,500 addresses/km^2^).

#### Difference in eventual vaccine uptake

**Figure 4:**
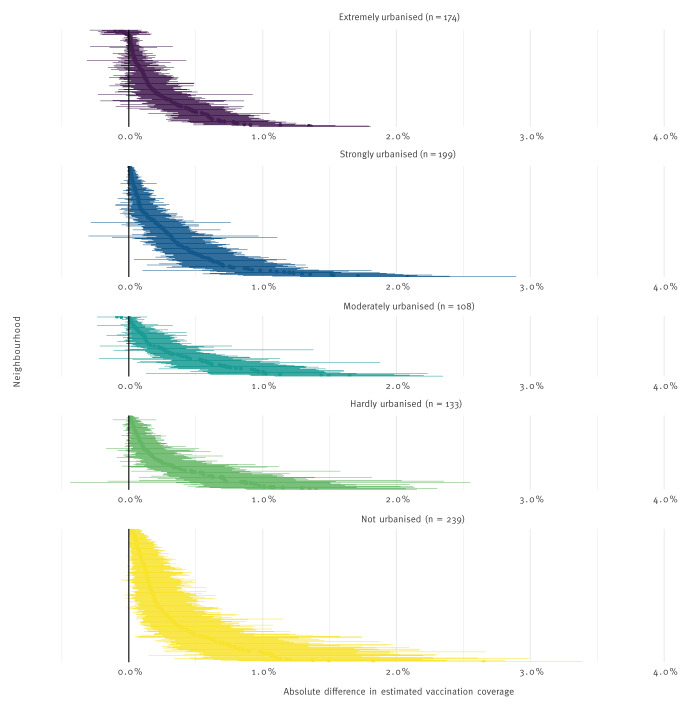
Difference in vaccine uptake in neighbourhoods with and without mobile vaccination units, stratified by level of urbanisation, the Netherlands, September–December 2021

Table 3 and Figure 4 present the relative difference in eventual vaccine uptake during the intervention period; it represents the difference in overall vaccine uptake between deployment and non-deployment of MV-units. The difference in eventual vaccine uptake over the study period varied from 0.33 percentage points (CI: 0.28–0.37) in rural neighbourhoods, to 0.22 percentage points in very urbanised neighbourhoods (CI: 0.18–0.26).

**Table 3:** Pooled relative differences, total difference in vaccine uptake in neighbourhoods, by level of urbanisation, the Netherlands, September–December 2021

Characteristics	Relative difference	95% CI
Not urbanised	0.33	0.28–0.37
Hardly urbanised	0.30	0.24–0.36
Moderately urbanised	0.35	0.28–0.42
Strongly urbanised	0.31	0.26–0.36
Extremely urbanised	0.22	0.18–0.26

CI: confidence interval.

Not urbanised (< 500 addresses/km^2^); hardly urbanised (500–999 addresses/km^2^); moderately urbanised (1,000–1,499 addresses/km^2^); strongly urbanised (1,500–2,499 addresses/km^2^); extremely urbanised (≥ 2,500 addresses/km^2^).

Not urbanised (< 500 addresses/km^2^); hardly urbanised (500–999 addresses/km^2^); moderately urbanised (1,000–1,499 addresses/km^2^); strongly urbanised (1,500–2,499 addresses/km^2^); extremely urbanised (≥ 2,500 addresses/km^2^).

Figure 4 presents the difference in vaccine uptake per neighbourhood, calculated by comparing the model outcomes with MV-units with the model without MV-units. In the most urbanised areas, the difference in vaccine uptake ranged from 0 to ca 1.5% percentage points, with the largest CI not reaching 2. In the hardly urbanised neighbourhoods, the difference in uptake ranged from 0 to almost 2, and the largest CI exceeded 2 percentage points. In the least urbanised neighbourhoods, we saw a difference in uptake of 0 in a few neighbourhoods, increasing in a somewhat convex manner to a difference between 1 and 2 on average, indication that in some neighbourhoods, the increase in vaccine uptake was near 2 percentage points. The differences between the different levels of urbanisation were similar. In lesser urbanised neighbourhoods, the effect of the MV-units appeared to be stronger than in the more urbanised neighbourhoods. These effects were statistically different.

## Discussion

This study, where national vaccination registration data were combined with data on MV-unit deployment, showed that vaccination rate increased in most neighbourhoods where MV-units were deployed in the autumn of 2021 during the COVID-19 pandemic as well as in the contiguous neighbourhoods. In the targeted neighbourhoods, the increase in vaccination rate varied between a factor 1.1 in urbanised neighbourhoods and a factor 14.5 in rural neighbourhoods. The increase in vaccine uptake over the complete intervention period ranged from 0.22 percentage points (95% CI: 0.18–0.26) in the most urbanised neighbourhoods to 0.33 percentage points (95% CI: 0.28–0.37) in rural neighbourhoods.

The seemingly modest effect on eventual vaccine uptake may conceal the relevance on the neighbourhoods the MV-units were active. Compared with the long mass vaccination campaign, the MV-units were deployed for only a short time in few locations. The absolute effect estimate was very small despite the large relative rate due to the short and late deployment of the MV-units. In our observations, we compared 7.7% neighbourhood-days with MV-units with 92.3% neighbourhood-days without MV-units. More proactive and long-term deployment is required to identify its potential to substantially contribute to reduction of social and geographical inequalities in vaccine uptake [24,25].

Subgroup analyses showed that in neighbourhoods with more than 5% voters for the RPP, the effect of an MV-unit was stronger, while in areas with more people with a non-western migration background, the effect was weaker. It is important to note though, that there were still positive effects of MV-unit deployment. It is relevant that MV-units were often combined with additional educational activities – although their intensity, cultural-sensitivity and mode of delivery could not be analysed in this study. In neighbourhoods with higher percentages of voters for the RPP party, the presence of a MV-unit appeared to be a reason, more often than for other subgroups, to get vaccinated. Mistrust in the vaccinating institutions and religious motives are reasons to doubt getting a vaccine [26]. Being able to interact face-to-face in the MV-units and the opportunity to get additional information may have been facilitators of increased trust and increased uptake for these persons. It may be that those activities more effectively addressed hesitancy among people with a Reformed Protestant background than among people with diverse migration backgrounds.

We were able to evaluate the impact of MV-units on national scale in real-life (rather than in a more artificial e.g. randomised controlled trial setting) over a 3-month period, combining intervention exposure data with national vaccination registries and neighbourhood characteristics. The flexible mixed-effects regression model shares information between neighbourhoods, resulting in a smaller effective number of parameters to be estimated. The amount of smoothing was automatically estimated by the amount of information in the data using RMLE. Because of the shrinkage of effect sizes (i.e. effect sizes are pulled towards the average neighbourhood), no correction for multiple testing was needed [24,25].

The most important limitation in our study is the fact we could not determine causality in the effect of MV-units on vaccine uptake since we were unable to conduct a randomised study (i.e. the deployment of MV-units was not random). To estimate the effect of the deployment of the mobile vaccination units, some assumptions were made that weaken conclusions regarding causality. Firstly, we assumed that individuals that chose to get vaccinated in a MV-unit would not have been vaccinated otherwise. In addition, neighbourhoods served as their own controls, meaning we assumed the baseline vaccination coverage would not change. Furthermore, other unobserved factors may have influenced the results and have affected the vaccine uptake in a way that is not captured in the model. While we recognise these assumptions, we believe our study provides valuable insights into the potential impact of MV-units on vaccine uptake as it addresses a critical gap in the literature and offers valuable real-world data that can inform public health strategies.

There are a number of less important limitations that are worth noticing. Firstly, the national vaccination registry CIMS only contains the 93% of vaccinated people who consented to be registered. If people with less trust in the government are also less likely to consent to registration of their vaccination, this may cause little distortion in the registered uptake. However, for people who decide to get vaccinated, but refuse to consent to registration, this may have resulted in an underestimation in the effect of MV-units. Therefore, there is an unknown chance that the reported effects are in fact underestimations. Secondly, our data contains all MV-units made available by the National organisation of municipal health services (GGD-GHOR), but in some regions, other types of temporary vaccination locations in areas where many people worked or lived were deployed. These data were not available to us, which may have had some influence on our effect estimates. A third limitation is that even though the analyses identified significant mean differences between neighbourhoods, the heterogeneity is still considerable. We were able to include several neighbourhood level confounders, but not differences in the additional education and outreach activities that were organised around MV-units [25,27]. Lastly, although the use of proportion of votes for the RPP as a proxy for vaccine hesitancy is supported by previous studies, this approach may not capture all aspects of vaccine hesitancy, as individual motivations could also influence both vaccination and voting behaviour.

## Conclusion

Mobile vaccination units are effective in increasing vaccination rates in areas where initial vaccine uptake is lagging. The effect of the deployment of MV-units greatly varied between neighbourhoods and was highest in rural neighbourhoods. Also, in urban neighbourhoods with a high proportion of people with a non-western migration background, the presence of MV-units increased the vaccination rate. Although it is unclear whether the effects of MV-units would have sustained if deployed on a larger scale and for a longer time, it appears that their brief and late deployment has limited their impact on national-level vaccine uptakes. The conditions under which MV-units are cost-effective remains to be evaluated.

## †Erratum

In the model written in pseudo-equation form, part of the equation was missing at the time of publication. This mistake was corrected on 23 August 2024.

## Supplementary Data

998000 Supplementary Material
