# Erratum for Euro Surveill. 2024;29(34)

**DOI:** 10.2807/1560-7917.ES.2024.29.35.240829e

**Published:** 2024-08-29

**Authors:** Eurosurveillance editorial team

**Affiliations:** 1 European Centre for Disease Prevention and Control (ECDC), Stockholm, Sweden

In the article entitled ‘Mobile vaccination units to increase COVID-19 vaccination uptake in areas with lower coverage: a within-neighbourhood analysis using national registration data, the Netherlands, September–December 2021’ by Lambooij et al., in the model written in pseudo-equation form, part of the equation was missing at the time of publication. This mistake was corrected on 23 August 2024.

