# Supplementary material for: Mobile vaccination units to increase COVID-19 vaccination uptake in areas with lower coverage: a within-neighbourhood analysis using national registration data, the Netherlands, September–December 2021
Source: Euro Surveill. 2024 Aug 22;29(34):2300503. doi: 10.2807/1560-7917.ES.2024.29.34.2300503 (PMC11367067; doi:10.2807/1560-7917.ES.2024.29.34.2300503)
Supplement: Supplementary Material [file 23-00503_LAMBOOIJ_Supplement.pdf]

Supplementary material

This supplementary material is hosted by Eurosurveillance as supporting information alongside the article " Mobile vaccination units to increase COVID-19 vaccination uptake in areas with lower coverage: a within-neighbourhood analysis using national registration data, the Netherlands, September–December 2021" , on behalf of the authors, who remain responsible for the accuracy and appropriateness of the content. The same standards for ethics, copyright, attributions and permissions as for the article apply. Supplements are not edited by Eurosurveillance and the journal is not responsible for the maintenance of any links or email addresses provided therein.

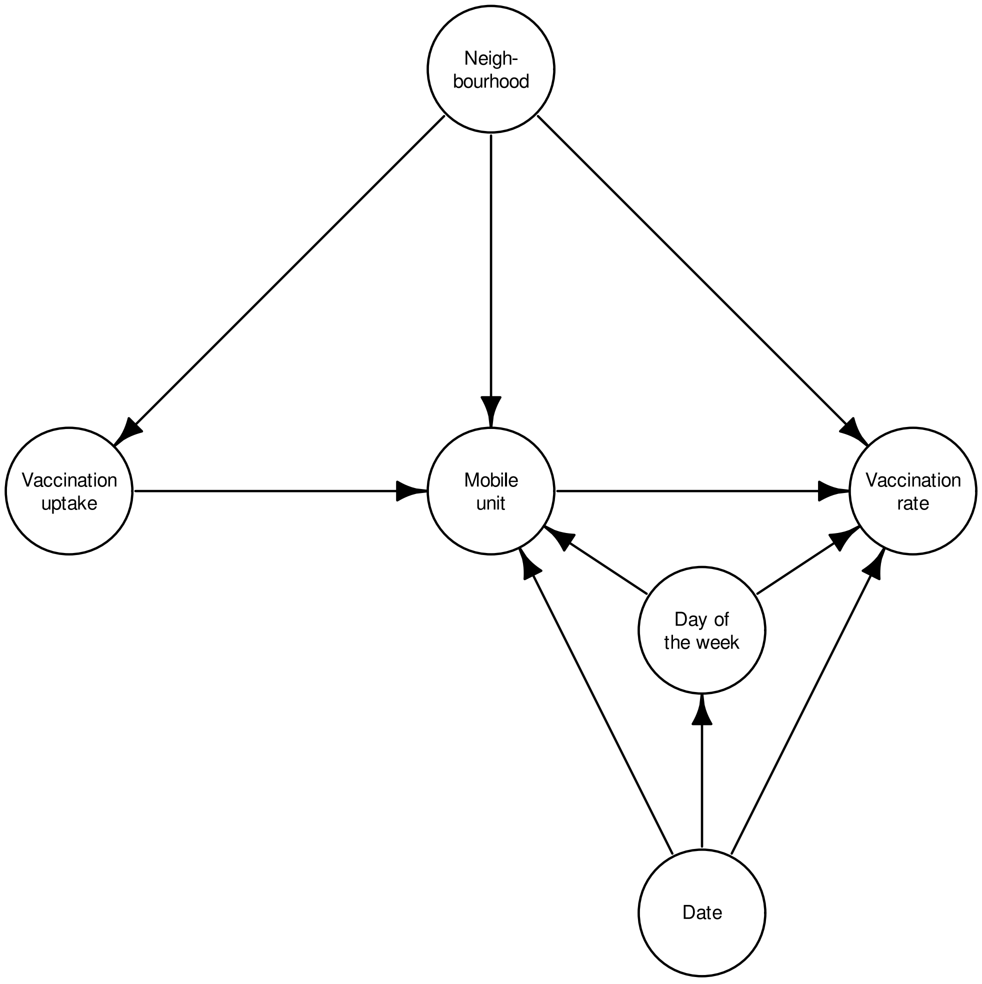

Figure S1. *Expected causal diagram leading to the daily vaccination rate. Variables are denoted by the vertices (circles), causal pathways by the edges (arrows).*

S2. Vaccination uptake per neighbourhood

Supplementary figure S2 shows the vaccination uptake per neighbourhood on September 1st, 2021. The map indicates geographical variation in vaccination uptake, where the vaccine uptake is lower in the Dutch Bible Belt area, an area with a high concentration of voters for right-wing Christian parties, stretching from the southwest through the centre of the country, to parts in the northeast. In all regions, vaccinations were less likely administered on Sundays, compared to other days of the week. This is the result of limited opening hours for many of the vaccination locations on Sundays. A timeline of the daily vaccine uptake in the 973 neighbourhoods can be found in supplementary file 4.

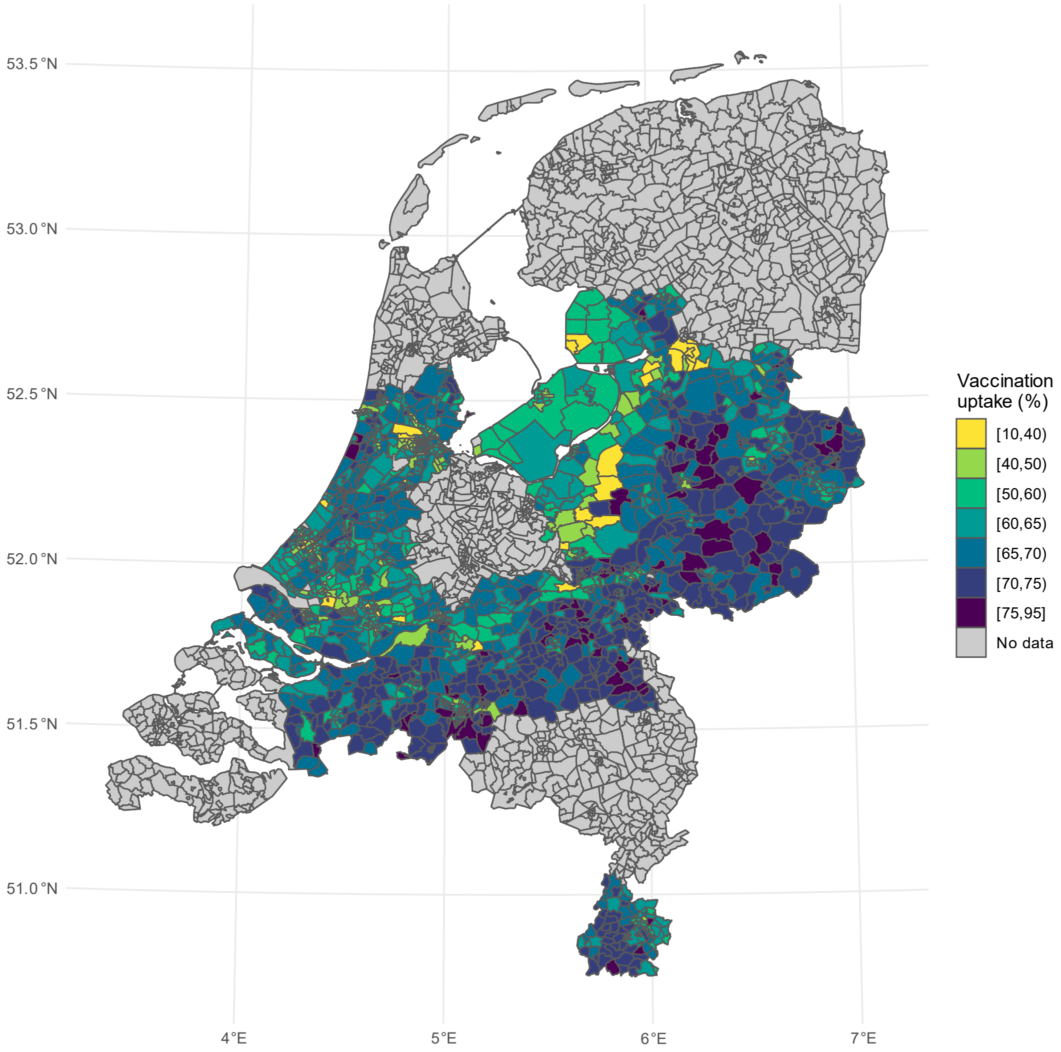

Figure S2. *Map showing the percentage of individuals that received at least one COVID-19 vaccination for all neighbourhoods within the 16 included municipal health regions on September 1st, 2021. The thick grey lines indicate the boundaries of the 25 municipal health regions in the Netherlands.*

S3. Presence of mobile vaccination units

Supplementary figure 3 shows a timeline of the presence of MV-Units by date and neighbourhood. The blue-coloured bars are dates when a unit was present in that neighbourhood, the yellow-coloured bar are dates when a neighbourhood was indicated as a contiguous neighbourhood. For most neighbourhoods, a unit was present for only one day.

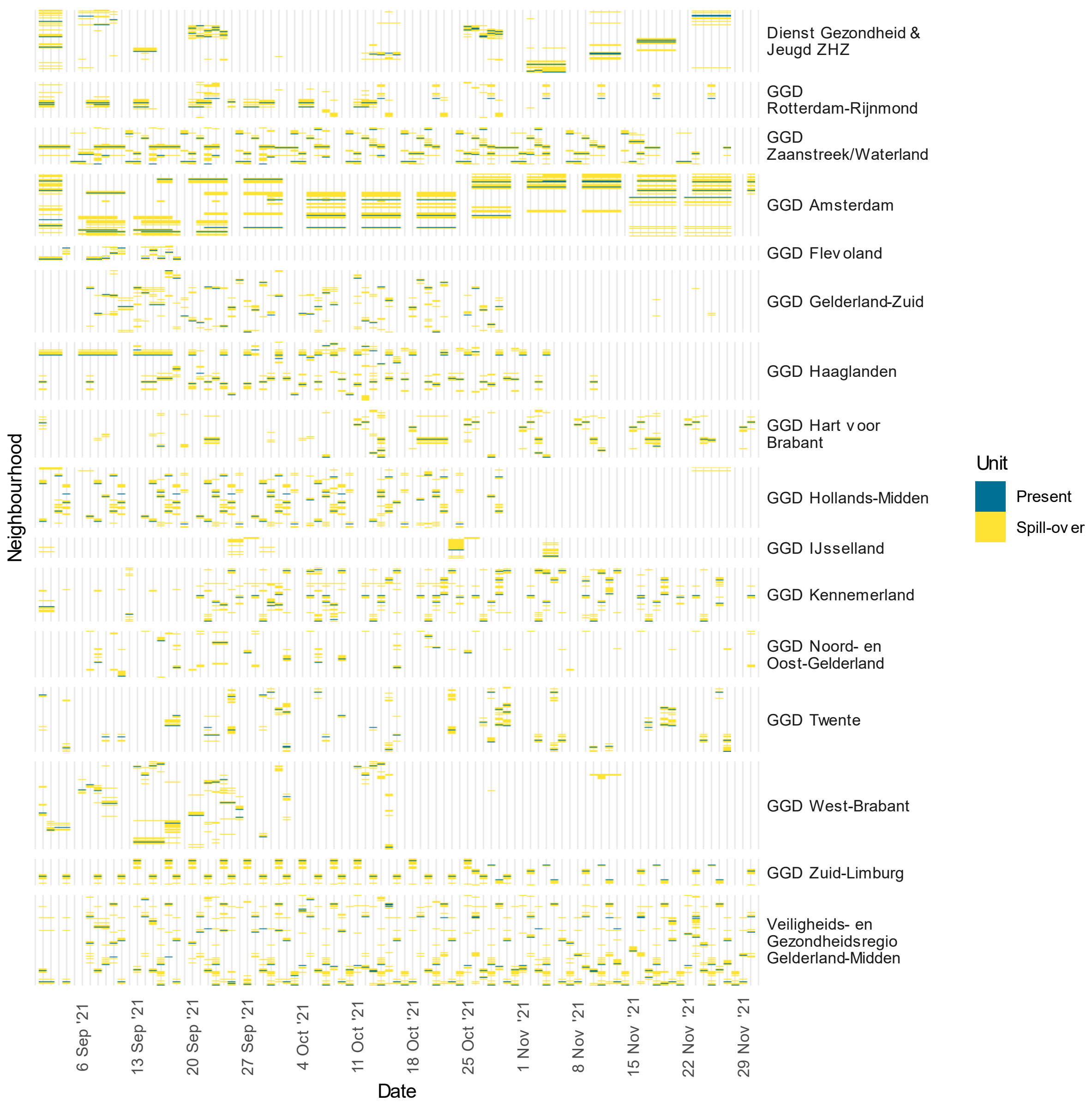

***Figure 3**. Timeline of the presence of MV-Units for the* 973 *neighbourhoods that are either targeted (present) or non-targeted (contiguous) (as in figure 3), stratified by municipal health region. Each horizontal line represents a neighbourhood, arranged by neighbourhood-code.*

S4. Vaccination rates over time

Supplementary figure 4 shows that vaccination rates varied over time with relatively higher rates in September and November. Furthermore, vaccinations were less likely administered on Sundays, compared to other days of the week.

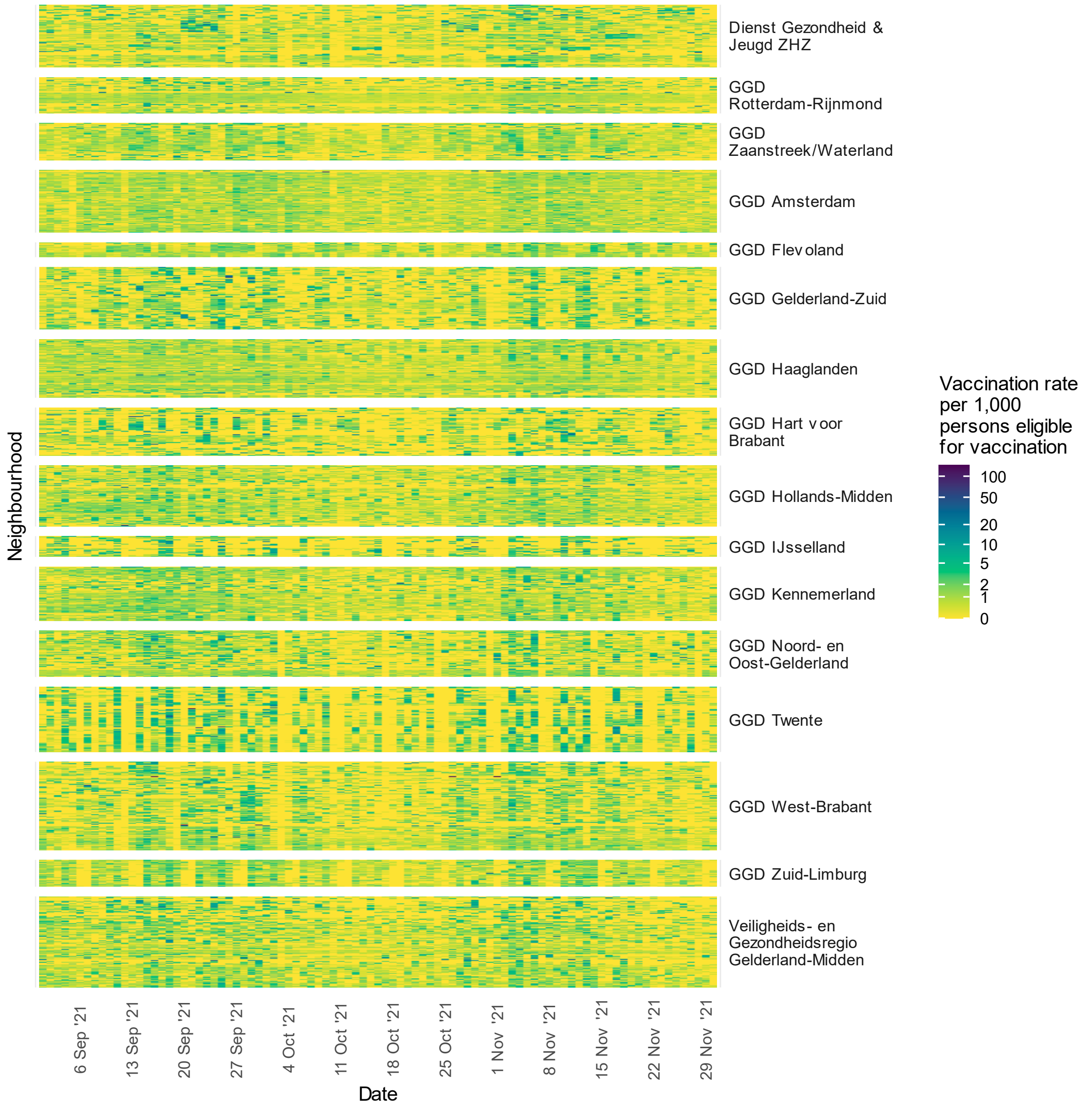

Figure S4. *Daily vaccination rates for the* 973 *neighbourhoods (as in figures 2 and 3) per 1,000 persons eligible for vaccination, stratified by municipal health region. Each horizontal line represents a neighbourhood, arranged by neighbourhood-code.*

S5. Relative rates and 95% confidence intervals for contiguous neighbourhoods

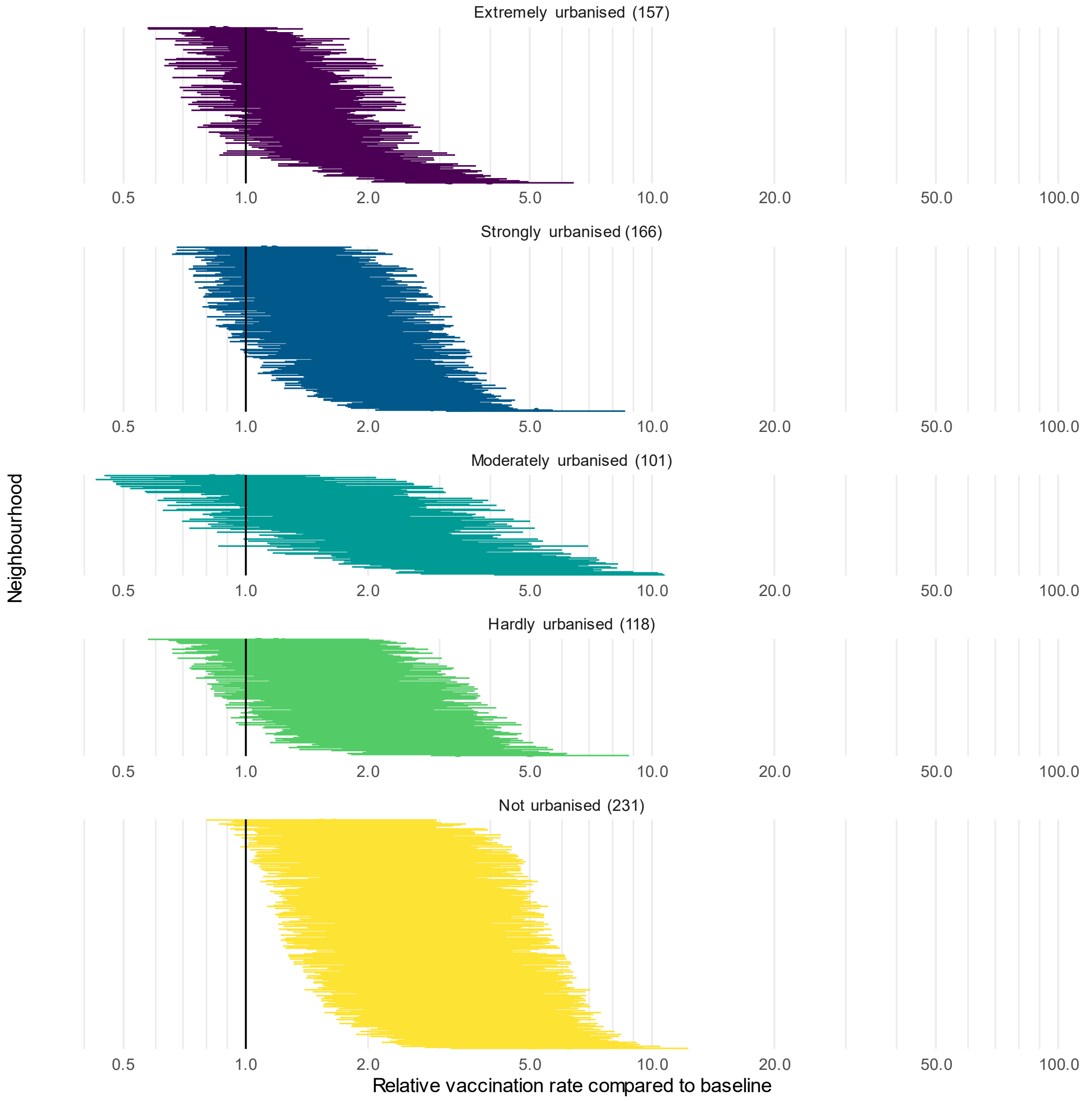

Figure S5. *Relative rates and 95% confidence intervals for contiguous neighbourhoods of daily vaccination rates on a day with a MV-unit deployed, compared to the same neighbourhood with no MV-unit deployed, stratified by level of urbanisation.*

S6. ***Differences in vaccination uptake***

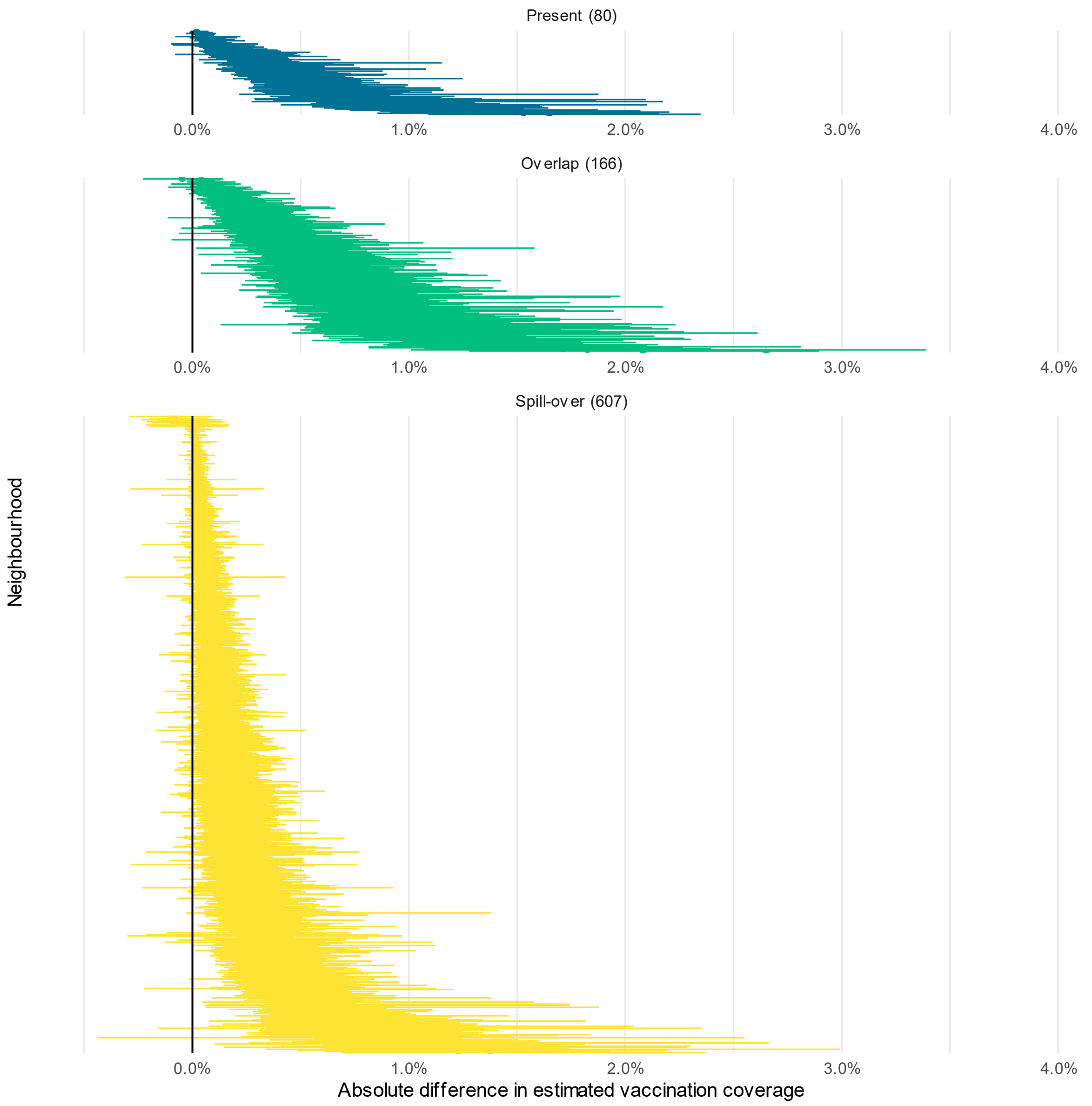

Figure S6*. Difference in vaccination uptake in target neighbourhoods, contiguous neighbourhoods and neighbourhoods with overlap of present and spill-over exposure at the end of the study period.*
